# Editorial: Spotlighting the interaction network of hub genes, molecules, and cells in the tumor immune microenvironment (TIME) and their contribution to malignant progression

**DOI:** 10.3389/fimmu.2024.1533290

**Published:** 2024-12-11

**Authors:** Jianpeng Li, Xuanbin Wang, Run Shi

**Affiliations:** ^1^ Department of Cardiology, Taizhou Second People’s Hospital, The Affiliated Taizhou Second People’s Hospital of Yangzhou University, Taizhou, China; ^2^ Laboratory of Chinese Herbal Pharmacology, Department of Pharmacy, Renmin Hospital, Hubei University of Medicine, Shiyan, China; ^3^ Department of Oncology, The First Affiliated Hospital of Nanjing Medical University, Nanjing, China

**Keywords:** tumor microenvionment, molecular subtyping, machine learning, precise medicine, cancer progression

The tumor immune microenvironment (TIME) is a complex and dynamic network that comprises diverse elements, including various cell types, extracellular matrix components, and secreted molecules. These components interact with each other and deeply influence malignant phenotypes and therapeutic responses (1). For example, cancer cells can evade each step of the cancer immunity cycle by interacting with various immune cells (2). They can induce the recruitment of immunosuppressive cells, such as regulatory T cells (Tregs) and myeloid-derived suppressor cells (MDSCs), which could inhibit the activation and function of cytotoxic cells like T cells and natural killer (NK) cells (3). Furthermore, some tumor-associated macrophages (TAMs) can develop into a pro-tumor and immunosuppressive phenotype in response to tumor-derived signals (4). Besides, TIME has an impact on genomic instability and angiogenesis, and these alterations can jointly attenuate the therapeutic efficacy, especially immunotherapy for cancer (5). Therefore, we established the Research Topic to encourage researchers to focus on and further investigate the complex interactions within the TIME and their implications for cancer progression and treatments.

The Research Topic includes seven original articles and three reviews with a wide range of diverse cancer types. Three original articles focused on the tumor microenvironment (TME) and relevant molecules in hepatocellular carcinoma (HCC) from different perspectives. Gong et al. identified distinct molecular subtypes associated with neutrophils in HCC, exhibiting significant differences in prognosis, clinical pathological characteristics, inflammation-related pathways, and immune-related features. Furthermore, the authors constructed a neutrophil-derived signature (NDS) to predict overall survival and efficacy of immunotherapy and chemotherapy for HCC patients using machine learning approaches. Additionally, Xu et al. reported two distinct m^6^A modification patterns based on the 23 m^6^A regulators, and the two m^6^A subtypes correlate with different clinical outcomes and biological features. Subsequently, they developed an m^6^A risk score model to improve survival prediction and estimation of drug responses for HCC patients. Moreover, Ouyang et al. conducted a comprehensive bioinformatics analysis to evaluate both the expression and mutation patterns of PANoptosis-related genes (PRGs) in HCC, and a PANoptosis risk model was constructed to offer a precise prediction of clinical outcomes and therapeutic sensitivity for HCC patients. The authors then performed experiments to validate the expression profiles and biological functions of their identified hub genes involved in the PANoptosis-related gene signature.

Despite different malignancies (osteosarcoma and chronic myeloid leukemia), the studies of Wu et al. and Zhong et al. shared similar ideas in investigating TME and implications for cancer treatment. They focused on differentially expressed genes (DEGs) and then identified different clusters with distinct immunological properties based on their expression profiles. They also commonly performed LASSO regression analysis to screen for key biomarkers of diagnosis or prognosis. Furthermore, sensitive drugs for specific subtypes or high-risk populations were investigated for precise treatment.

As regards immunosuppression, Bi et al. found that high expression of CDKL3 in esophageal cancer (ESCA) was not only associated with poor prognosis but also negatively correlated with the abundance of tumor-infiltrating immune cells and anti-tumor immune response. These findings suggest CDKL3 as an immunosuppressive molecule in the TME of ESCA. The authors also reported that the knockdown of CDKL3 in ESCA cells could inhibit autophagy induction and M2 macrophage polarization. Hypoxic TME is also a critical factor in the progression and outcome of solid cancers (6). To explore its influence on tumor progression and therapy outcome, Zhang et al. utilized Lasso regression to analyze transcriptomic data of patients with colorectal cancer (CRC) and identified seven robust hypoxia-associated genes. Based on these genes, the authors further established a novel prognostic score for CRC called the hypoxia-related prognosis score (HPS), and they found that HPS is significantly related to different extracellular matrix compositions, various immune cell infiltration, and suppressive immune response. The two articles suggest that some immunosuppressive factors involved in TME play important roles in shaping tumor progression, therapeutic resistance, and patient outcomes in solid cancers. These findings highlight the complexity of the TME in modulating immune responses and reveal the potential of targeting these factors to improve the therapy efficacy.

Overall, all the above-mentioned seven original articles regarding the identification of distinct molecular subtypes, immune-regulatory genes or molecules, and prognostic biomarkers in diverse cancer types or subtypes offer valuable insights into tumor progression and therapeutic sensitivity, which shows a promising way for personalized treatment strategies for cancer patients.

Li et al. focused on plasmablastic lymphoma (PBL), a rare but aggressive non-Hodgkin lymphoma. They comprehensively summarized the current knowledge on the epidemiology, molecular profiles, clinical and pathological features, differential diagnosis, treatment strategies, prognostic factors, and potential novel therapeutic approaches in PBL patients. This review highlights the fact that, despite developments in treatment strategies such as intensive chemotherapy, targeted therapies, and immunotherapy, the prognosis of PBL remains poor. Therefore, there is an urgent need for further exploration of PBL’s biological characteristics and the development of more effective targeted therapeutic approaches. Another review from Guo et al. summarized five cellular composition modules by integrating the cellular (sub)types, phenotypes, and functions in the TME of pancreatic ductal adenocarcinoma (PDAC). Furthermore, the authors pointed out that cross-module regulations are determinants of the immunosuppressive TME in PDAC, and highlighted TME-targeted strategies that potentially improve PDAC therapy. In addition, Gunes et al. reviewed the current knowledge of the expression of signaling lymphocytic activation molecule family (SLAMF) receptors in solid tumors and tumor-infiltrating immune cells and summarized their associations with patient outcomes. The authors also discussed the therapeutic potential of targeting SLAMF receptors to improve outcomes of cancer therapy in solid tumors. Thus, a better understanding of the interactions between SLAMF receptors and TME components may contribute to the development of interventions that can reprogram the TME into a more favorable environment to enhance the efficacy of cancer therapy such as immunotherapy.

In summary, these studies contributed by diverse authors in this Research Topic highlight the important roles of TIME in cancer progression and therapeutic resistance. We believe these findings could show a promising way for personalized strategy to reprogram the TIME for improving cancer management.
